# Metabolomics study reveals the potential evidence of metabolic reprogramming towards the Warburg effect in precancerous lesions

**DOI:** 10.7150/jca.54252

**Published:** 2021-01-10

**Authors:** Xun Chen, Chen Yi, Man-Jun Yang, Xueqi Sun, Xubin Liu, Hanyu Ma, Yiming Li, Hongyu Li, Chao Wang, Yi He, Guanhui Chen, Shangwu Chen, Li Yu, Dongsheng Yu

**Affiliations:** 1 Guanghua School of Stomatology, Guangdong Provincial Key Laboratory of Stomatology, Sun Yat-sen University, Guangzhou 510055, People's Republic of China.; 2 Center for Proteomics and Metabolomics, State Key Laboratory of Biocontrol, Guangdong Province Key Laboratory for Pharmaceutical Functional Genes, School of Life Sciences, Sun Yat-sen University, Guangzhou 510006, People's Republic of China.; 3 Department of Pathology, the First Affiliated Hospital, Sun Yat-sen University, Guangzhou 510080, People's Republic of China.; 4 Guangdong Key Laboratory of Pharmaceutical Functional Genes, MOE Key Laboratory of Gene Function and Regulation, State Key Laboratory for Biocontrol, Department of Biochemistry, School of Life Sciences, Sun Yat-sen University, Guangzhou 510275, People's Republic of China.

**Keywords:** metabolic reprogramming, precancerous lesions, metabolomics, the Warburg-like effect, glycolytic enzymes

## Abstract

**Background:** Most tumors have an enhanced glycolysis flux, even when oxygen is available, called the aerobic glycolysis or the Warburg effect. Metabolic reprogramming promotes cancer progression, and is even related to the tumorigenesis. However, it is not clear whether the observed metabolic changes act as a driver or a bystander in cancer development.

**Methods:** In this study, the metabolic characteristics of oral precancerous cells and cervical precancerous lesions were analyzed by metabolomics, and the expression of glycolytic enzymes in cervical precancerous lesions was evaluated by RT-PCR and Western blot analysis.

**Results:** In total, 115 and 23 metabolites with reliable signals were identified in oral cells and cervical tissues, respectively. Based on the metabolome, oral precancerous cell DOK could be clearly separated from normal human oral epithelial cells (HOEC) and oral cancer cells. Four critical differential metabolites (pyruvate, glutamine, methionine and lysine) were identified between DOK and HOEC. Metabolic profiles could clearly distinguish cervical precancerous lesions from normal cervical epithelium and cervical cancer. Compared with normal cervical epithelium, the glucose consumption and lactate production increased in cervical precancerous lesions. The expression of glycolytic enzymes LDHA, HK II and PKM2 showed an increased tendency in cervical precancerous lesions compared with normal cervical epithelium.

**Conclusions:** Our findings suggest that cell metabolism may be reprogrammed at the early stage of tumorigenesis, implying the contribution of metabolic reprogramming to the development of tumor.

## Introduction

Metabolic reprogramming is one of the hallmarks of cancer. Metabolic changes in cancer involve many aspects of metabolism, such as the deregulation of glucose and amino acid uptake and consumption, and the application of glycolysis/tricarboxylic acid (TCA) cycle intermediates in biosynthesis 1. The survival and growth of cancer cells are more dependent on glycolysis, even if there is sufficient oxygen 2. This type of aerobic glycolysis, discovered nearly a century ago, is now known as the Warburg effect 3, 4. Glycolysis is a basic metabolic pathway of carbohydrates, in which one glucose molecule is converted into two molecules of pyruvate. Hexokinase (HK), phosphofructokinase (PFK), and pyruvate kinase (PK) are key enzymes in the control of cellular glycolytic flux. Pyruvate can be reduced to lactate by lactate dehydrogenase (LDH) under hypoxia. Glucose transporters (GLUTs) transport glucose into cells, whilst monocarboxylate transporters (MCTs) facilitate the export of lactate into extracellular space. Thus, these enzymes and proteins are involved in regulating the rate of glycolysis. Hypoxia-inducible factor-1α (HIF-1α) is a transcription factor that transactivates the expression of GLUTs and many glycolytic enzymes, promoting glycolysis in tumors. Although most cancer cells rely on high glycolysis rates, metabolic reprogramming as a cause of cancer development or a consequence of tumorigenesis remains an open question.

The development of cancer is a complex process that is not fully understood. Precancerous lesions/stages are often observed in the development of some cancers, such as squamous cell carcinoma. For example, oral lichen planus, leukoplakia, and erythroplakia are considered as oral potentially malignant disorders that may develop into oral squamous cell carcinoma (OSCC) 5. Human papillomavirus (HPV) infection can cause cervical squamous intraepithelial lesions (SIL), which are cervical precancerous lesions 6. Low-grade squamous intraepithelial lesions (LSIL) reflect morphologic changes of transient HPV infection with a high rate of regression, whilst high-grade squamous intraepithelial lesions (HSIL) represent persistent high-risk HPV infection and viral integration with a significant rate of progression to invasive carcinoma 7. Although a few studies have investigated metabolic alterations in precancerous lesions, it is unclear whether metabolic reprogramming occurs in precancerous lesions 8. Reprogrammed metabolism in precancerous lesions probably indicates that such reprogramming plays an important role in the development of cancer.

Metabolomics aims to characterize small molecules in biological samples and is widely applied in many aspects of cancer research, including cancer pathophysiology, biomarker discovery, and therapeutic response 9, 10. Mass spectrometry (MS) and nuclear magnetic resonance (NMR) spectroscopy are common methods used in metabolomics. In this study, the metabolic changes in oral precancerous cells and cervical precancerous lesion tissues were analyzed by mass spectrometry. The expression of glycolysis-related enzymes and proteins in cervical precancerous lesions was examined by RT-PCR and Western blot analysis. The results will be helpful to provide evidence for metabolic reprogramming in precancerous stages and its role in the development of cancer.

## Materials and methods

### Cell culture

This study involved a normal human oral epithelial cell (HOEC) (Bnbio, Beijing, China), a dysplastic oral keratinocyte (DOK) (Sgdbio, Shanghai, China), and three tongue squamous cell carcinoma cell lines Tca8133 (Shuaiyue, Shanghai, China), SCC-9 and SCC090 (Sgdbio, Shanghai, China). Cells were maintained in RPMI-1640 medium (DOK, Tca8133), DMEM (HOEC, SCC-9) and McCoy's 5A (SCC090) with 10% FBS (GIBCO, Australia), 100 U/mL penicillin G and 100 μg/mL streptomycin in a humidified atmosphere of 5% CO_2_ at 37°C.

### Tissue samples

In total, for this study 80 frozen biopsies, including 24 normal cervical epithelial tissues, 13 low-grade squamous intraepithelial lesions (LSIL), 22 high-grade squamous intraepithelial lesions (HSIL) and 21 squamous cell carcinoma (SCC) tissues, were obtained from the Department of Pathology, the First Affiliated Hospital, Sun Yat-sen University. All specimens were diagnosed by histopathology and stored at -80°C. Forty-eight specimens, including 15 normal cervical epithelia, 6 LSIL, 14 HSIL and 13 SCC, were used for real-time reverse transcription-polymerase chain reaction (RT-PCR), and 32 specimens, including 9 normal cervical epithelia, 7 LSIL, 8 HSIL and 8 SCC, for Western blot analysis. The research involving human tissues was approved by the Medical Ethics Review Board of the First Affiliated Hospital, Sun Yat-sen University, in accordance with the guidelines for the protection of human subjects. All patients signed consent forms.

### RNA extraction and quantitative real-time RT-PCR

Biopsy samples were rapidly cryo-sectioned and the tissues of normal squamous epithelia, SIL and SCC were isolated under microscopy. Tissues of about 20 mg or adherence cells in a 25-cm^2^ culture flask were incubated with 1 mL TRIzol reagent (Invitrogen Life Technologies, Carlsbad, CA, USA) for RNA isolation in accordance with the manufacturer's instructions. RNAs were treated with gDNA Eraser to remove the genomic DNA and reverse-transcribed with random hexamer primers using PrimeScript RT Enzyme Mix I (PrimeScript^TM^ RT reagent Kit with gDNA Eraser, TaKaRa, Shiga, Japan). Real-time PCR was performed in a 20 μL reaction volume with SYBR^®^ Premix Ex Taq II (Tli RNaseH Plus, TaKaRa, Shiga, Japan) and the ABI PRISM^®^7900 system (ABI). Reactions were processed in triplicate, and the threshold cycles and relative fold differences were calculated with 2^-ΔΔCt^.

### Western blot

Proteins were extracted by a total protein extraction kit and quantified by a BCA protein assay kit (CoWin Biotech, Beijing, China). About 20 μg of proteins were separated in 10% sodium dodecyl sulfate polyacrylamide gel electrophoresis (SDS-PAGE) gel and transferred to polyvinylidine difluoride membranes (Millipore, Bedford, MA, USA). The membranes were blocked with 5% skimmed milk powder in TBST buffer and then incubated with primary antibodies (anti-Hexokinase II antibody, 1:1000 dilution, Abcam; anti-Fructose 6 Phosphate Kinase antibody, 1:1000 dilution, Abcam; PKM2 (D78A4)XP® Rabbit mAb, 1:1000 dilution, Cell Signaling Technology; LDHA (C4B5) Rabbit mAb, 1:1000 dilution, Cell Signaling Technology; anti-Glucose Transporter GLUT1 antibody, 1:5000 dilution, Abcam; β-actin Rabbit mAb, 1:1000 dilution, Cell Signaling Technology) at 4°C overnight. After being washed, membranes were incubated with corresponding horseradish peroxidase-conjugated secondary antibodies (anti-rabbit IgG or anti-mouse IgG, HRP-linked antibody, Cell Signaling Technology, Danvers, MA, USA), and signals were visualized by the enhanced chemoluminescence method (Millipore, Bedford, MA, USA). The relative expression levels of proteins were quantified by ImageJ software with β-actin as the loading control.

### Gas chromatography-mass spectrometry (GC-MS)

GC-MS was used to detect the metabolic changes in several oral cells, fresh tissues of normal cervical epithelium, LSIL, HSIL, and SCC.

### Sample preparation and derivatization

The HOEC, DOK, SCC-9, SCC090 and Tca8113 cells were cultured to confluence in a petri dish and 2 × 10^6^ cells were collected as previously described, with some modifications 11. Briefly, cells were rinsed with distilled saline and then quenched thoroughly with 1 mL -20°C cold methanol (Sigma-Aldrich, St. Louis, MO, USA). The sediments were isolated by centrifugation with 6000 × *g* at 4°C for 10 min. The metabolites were extracted with 1 mL methanol by means of an ultrasonic cell disruptor (Scientz-950E, Ningbo, China) at a vibrational frequency of 360 W/40 kHz for 5 min. Then, a quantity of 10 µL ribitol (0.1 mg/mL) was added to each sample tube as an internal quantitative standard. The supernatant was harvested by centrifugation at 12,000 × *g* for 10 min and concentrated in a rotary vacuum centrifuge device (LABCONCO). The dried polar extracts were used for metabolite derivatization in GC-MS analysis. The dried residue was dissolved in 100 µL methoxyamine pyridine solution (20 mg/mL) and incubated at 37°C for 120 min in an incubator shaker. The mixture was treated with 100 µL N,O-Bis(trimethylsilyl)trifluoroacetamide (TMSTFA) with 1% trimethylchlorosilane (TMCS) and incubated at 37°C for 30 min. Every experiment was repeated by four biological replicates. In the case of biopsy samples, tissues of about 20 mg were homogenized in liquid nitrogen and supplemented with 1 mL methanol.

### GC-MS detection

The derivatized sample of 1 μL was injected into a HP-5MS column (Agilent Technologies, 30 m × 250 μm × i.d. 0.25 μm) by splitless injection, and analysis was carried out by Agilent 7890A GC equipped with an Agilent 5975C VL MSD detector (Agilent Technologies, Santa Clara, CA, USA). The initial temperature of the GC oven was held at 85°C for 5 min, followed by an increase to 280°C at a rate of 15°C per min, holding for 5 min, and increasing to 310°C at a rate of 20°C per min. Helium was used as the carrier gas, and flow was kept constant at 1 mL per min. The MS was operated at a range of 50-600 m/z.

### Spectrum processing for GC-MS

The deconvolution and calibration of the acquired mass spectra were performed with AMDIS (Agilent OpenLAB CDS ChemiStation C.01.01). To avoid false positives, we excluded peaks with a signal-to-noise ratio (S/N) lower than 30 12, and removed the artifact peaks by comparison with the blank samples. Metabolites were identified by retrieval of their mass spectra in the NIST 2011 (National Institute of Standards and Technology, USA) library and GMD 2011 (Golm Metabolome Database, Potsdam, Germany) according to the following criteria: match value ≥750, reverse match value ≥800 and a probability ≥60% 13. The relative peak area value of ribitol was taken as the internal standard for the calculation of metabolite abundance. This data array file can be used for subsequent multivariate statistical analyses.

### Bioinformatics analysis

Data transformation and manipulation were done in Excel. The differences in the metabolite contents between the two groups were compared by Analysis of Variance (ANOVA, α = 0.01) with SPSS 23.0 (IBM, USA). A multivariate statistical analysis of the metabolomic data was further performed via the MetaboAnalyst online Web site (www.metaboanalyst.ca/) 14-16. Z-score analysis scaled each metabolite according to a reference distribution. Z-score was calculated according to the formula, 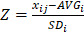, in which x_ij_, AVG_i_ and SD_i_ represented the metabolites' peak area, average of the control group and standard deviation of the control group, respectively. A hierarchical cluster analysis (HCA) was performed with the distance matrix calculated by the Euclidean method. Principal component analysis (PCA) and partial least squares-discriminant analysis (PLS-DA) were conducted for investigation of the relationships among the test samples. Based on the PLS-DA analysis, the compounds whose weight values of variable importance in the projection (VIP) were greater than 1 were filtered and shown in a scatter plot. For pathway enrichment analysis, the MetaboAnalyst online platform (www.metaboanalyst.ca/) 14-16 was used to determine the metabolic pathway of the metabolites showing differences between the test groups. The -log(*P*) value and a value reflecting the impact of each metabolic pathway were calculated by a hypergeometric test, and the metabolic pathways with *P* < 0.05 were retained. Prism v5.01 (GraphPad, La Jolla, CA, USA) was used to draw the histogram and the scatter plot.

## Results

### Metabolomic profiling of human oral precancerous cells

**Figure 1:**
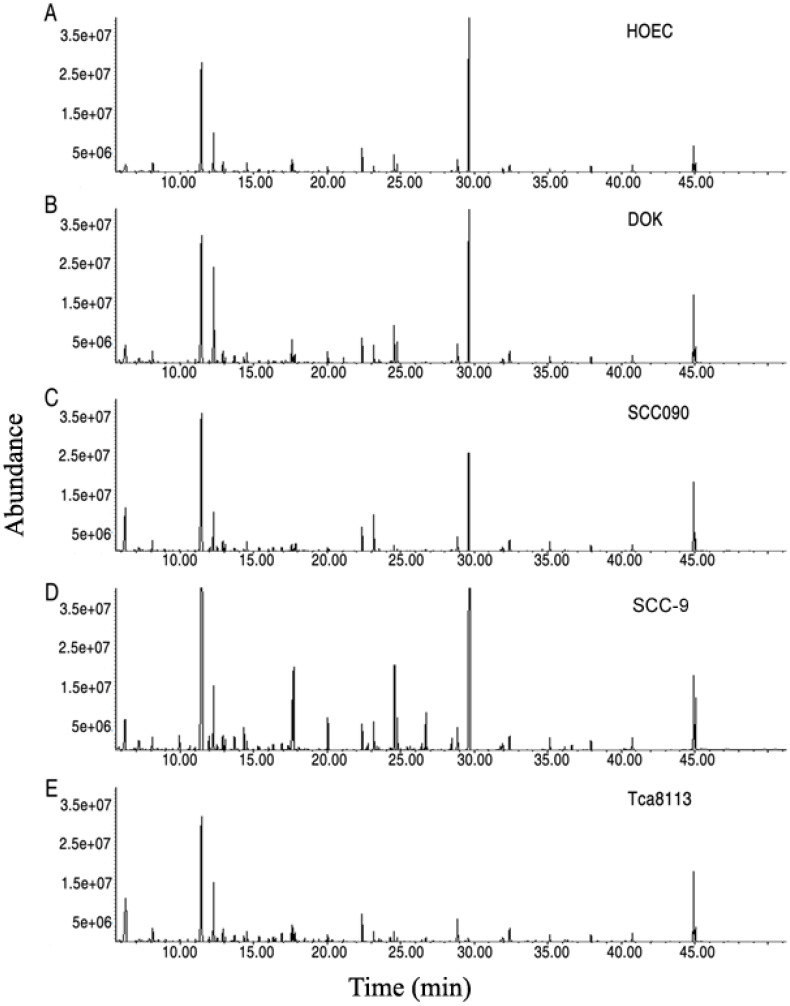
Metabolomics profiles of oral cell lines HOEC, DOK, SCC090, SCC-9 and Tca8113. Representative total ion current chromatograms are shown (A-E). X-axis, retention time (min); Y-axis, abundance.

For exploration of the metabolic alterations in precancer, a DOK cell, a precancerous cell established from human dysplastic oral mucosa 17, together with HOEC, a normal human oral epithelial cell and three oral cancer cells (SCC-9, SCC090 and Tca8113) were first subjected to GC-MS analysis. In total, 240 aligned individual peaks were obtained from each sample (Figure 1). After removal of the internal standard ribitol and any known artificial peaks and integration of the same compounds, 115 metabolites with reliable signals were identified in each sample. Four samples of each cell line, with two technical repeats, were examined, yielding a total of 40 data sets. The correlation coefficient between technical replicates varied between 0.9799 and 0.9999, demonstrating good reproducibility of the data (Figure S1 A). According to annotation in KEGG (http://www.kegg.jp/) and NCBI PubChem (https://pubchem.ncbi.nlm.nih.gov/), the metabolites were classified into five categories, including 25.22% carbohydrates, 28.70% amino acids, 17.39% nucleotides, 6.96% fatty acids and 21.74% other compounds (Figure S1B).

### Metabolic discrimination of DOK and HOEC cells

**Figure 2:**
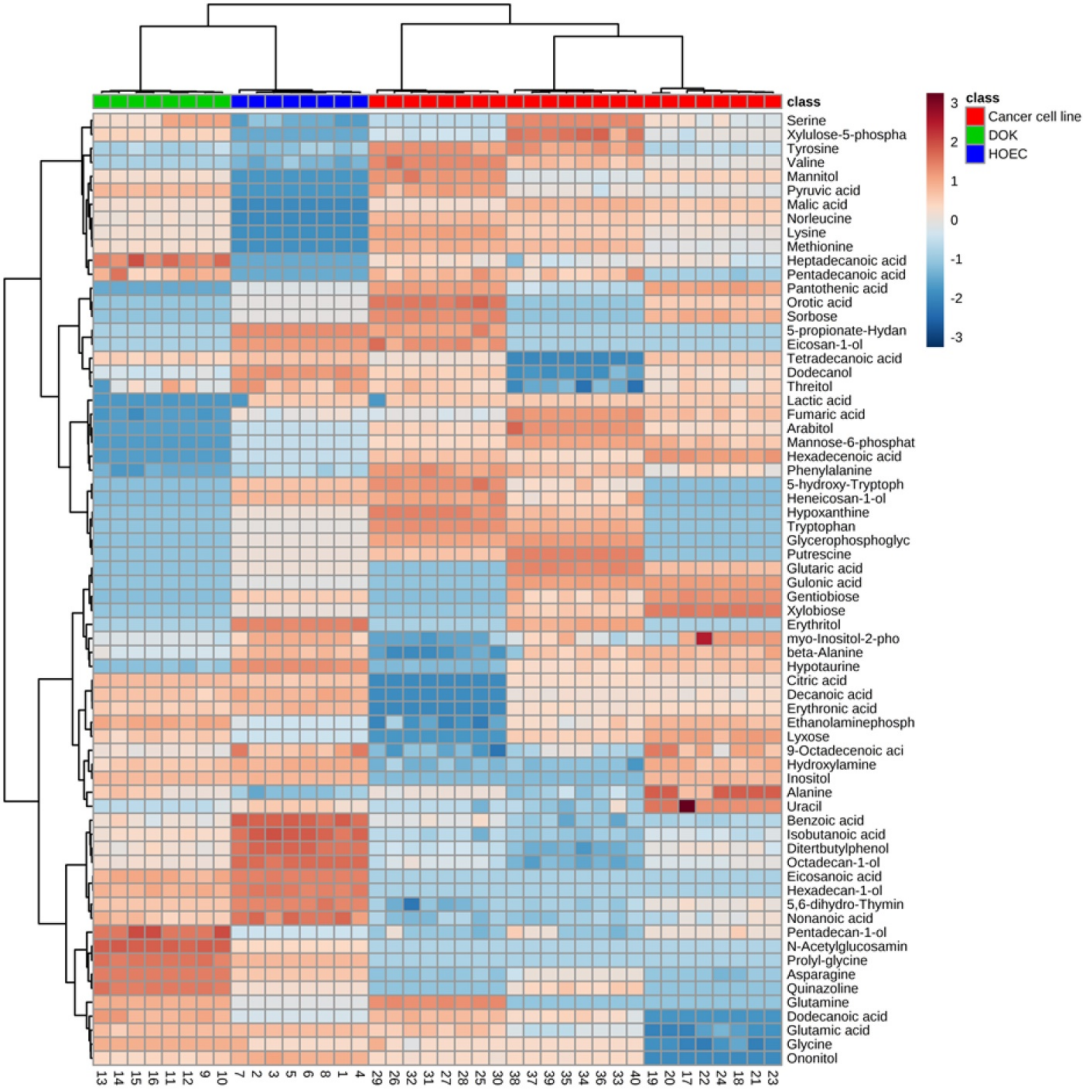
Differential metabolomics profiles of normal human oral epithelial cells (HOEC), dysplastic oral keratinocyte (DOK) and oral cancer cell lines SCC090, SCC9 and Tca8113. The heatmap shows 73 differential metabolites. Wine red and pewter indicate increased and decreased metabolites relative to the median metabolite levels, respectively (see color scale).

We wanted to know which metabolic biomarkers can distinguish these oral cells. ANOVA and a permutation test were used to determine the differential abundance of metabolites in different oral cell lines. Seventy-three significant metabolites, presented as a heatmap of hierarchical cluster analysis (HCA) (Figure 2), were identified in these cells (*P* < 0.01), which corresponded to the greatest false discovery rate (FDR) of phosphoric acid (0.01301). Oral cancer cells were clearly separated from DOK and HOEC cells.

**Figure 3:**
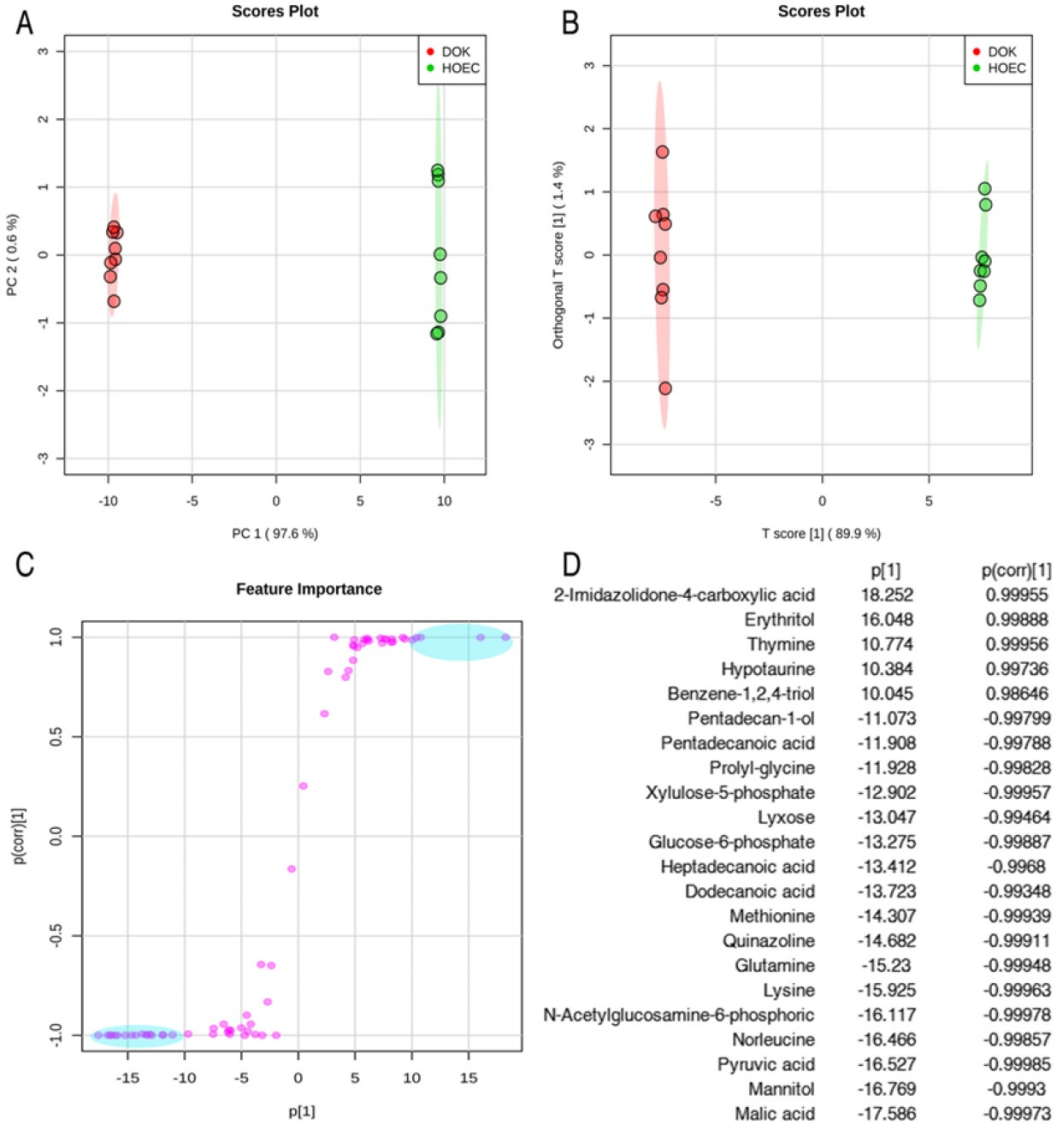
Identification of metabolomic candidates. PCA analysis of metabolites of HOEC and DOK cells (A). Each dot represents the technological replicate analysis of samples in the plot. PC1 and PC2 used in this plot explain 98.2% of the total variance, which allows for confident interpretation of the variation. Orthogonal partial least squares discriminant analysis (OPLS-DA, R2X=0.899, R2Y=0.999, Q2=0.999) (B). Score plot drawn with OPLS-DA (C). The crucial metabolomic candidates, shown in the light blue oval box (C), were selected by the weight absolute values of p^1^ and p(corr)^1^, which are more than 10 and 0.9, respectively (D).

In order to identify the candidate metabolites discriminating normal human oral epithelial cells (HOEC) and oral precancerous cells (DOK), we conducted unsupervised and supervised pattern discriminant analyses by using the principal component analysis (PCA) and orthogonal partial least squares discriminant analysis (OPLS-DA). PC1 (97.6%) and PC2 (0.6%) of PCA (Figure 3A) and Component 1 (T score 1 = 89.9%) and Component 2 (orthogonal T score 1 = 1.4%) of OPLS-DA (R2X=0.899, R2Y=0.999, Q2=0.999) separated the samples into two colonies (Figure 3B). PC1 and Component 1 clearly separated the DOK cells from HOEC cells (Figure 3A, 3B). Discriminating variables are shown as an S-plot (Figure 3C) when cut-off values of covariance p^1^ and correlation p(corr) were set as greater than or equal to 10 and 0.9, respectively. Twenty-two candidate metabolites, highlighted in blue oval boxes (Figure 3C), were identified through the screen of component p^1^ and p(corr)^1^, whose weight values are shown in Figure 3D.

**Figure 4:**
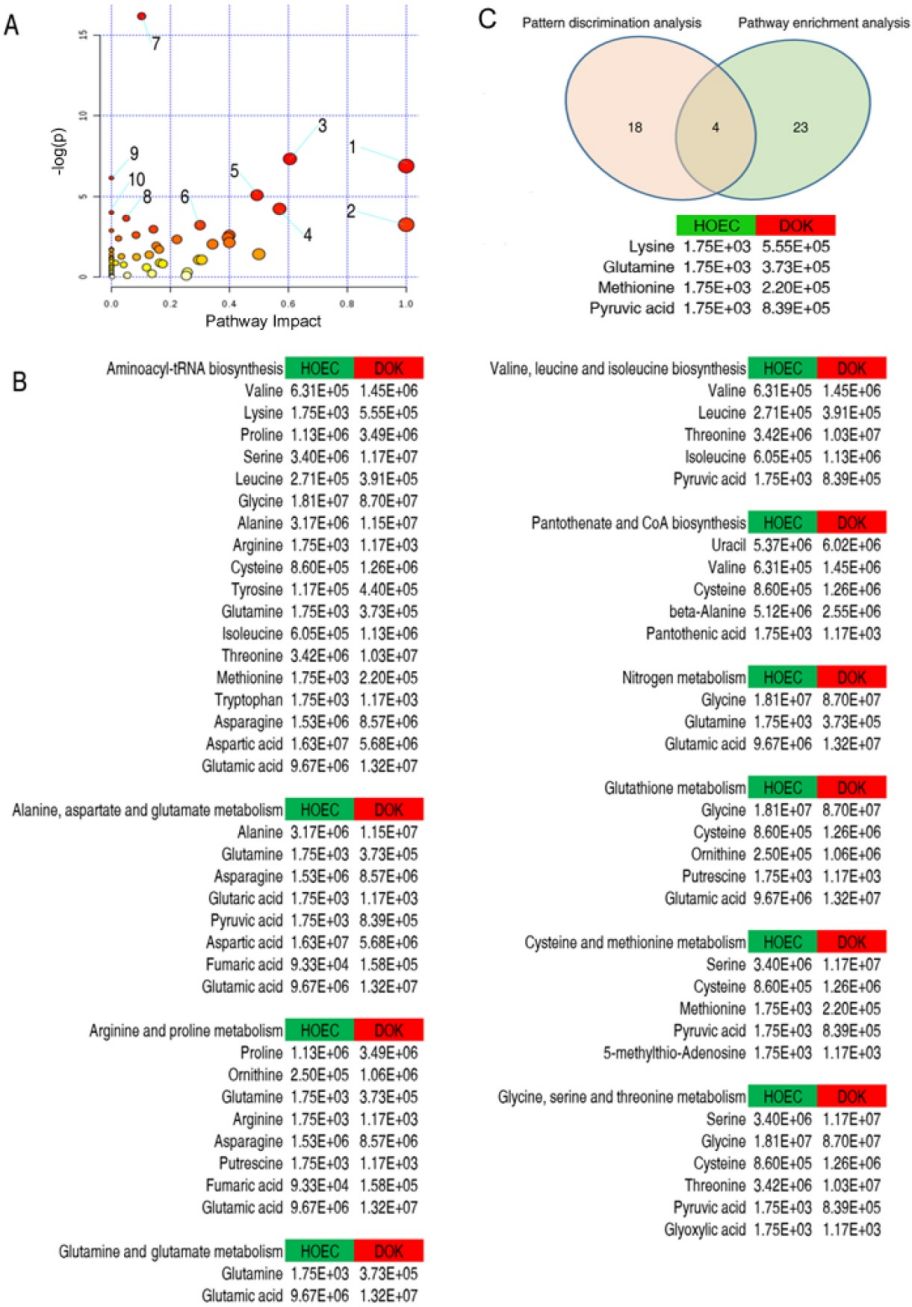
Enrichment analysis of metabolic pathways and its combination with pattern discrimination analysis. Significantly enriched pathways were selected to be plotted (A). From 1 to 10 represent glutamine and glutamate metabolism; valine, leucine and isoleucine biosynthesis; alanine, aspartate and glutamate metabolism; glycine, serine and threonine metabolism; arginine and proline metabolism; cysteine and methionine metabolism; aminoacyl-tRNA biosynthesis; glutathione metabolism; pantothenate and CoA biosynthesis; and nitrogen metabolism, respectively. The relative abundance of each metabolite is shown (B). Integration of metabolite pathway enrichment analysis and pattern discrimination analysis identified 4 crucial differential biomarkers responsible for the phenotype of DOK (C).

Identification of enriched metabolic pathways is important for understanding the metabolomic alterations in the development of cancer. Metabolite pathway enrichment analysis revealed that 10 metabolic pathways were enriched in HOEC and DOK cells (Figure 4A), which were sorted by their impact values. It is particularly interesting that amino acid metabolism may play an important role in the development of HOEC to DOK (Figure 4B).

Eventually, 4 crucial differential biomarkers (pyruvate, glutamine, methionine and lysine) were identified between HOEC and DOK by the integration of 27 significant metabolites from metabolite pathway enrichment analysis and 22 metabolomic candidates from pattern discrimination analysis, which may be related to the development of DOK (Figure 4C).

### Metabolomic profiling of human cervical precancerous tissues

**Figure 5:**
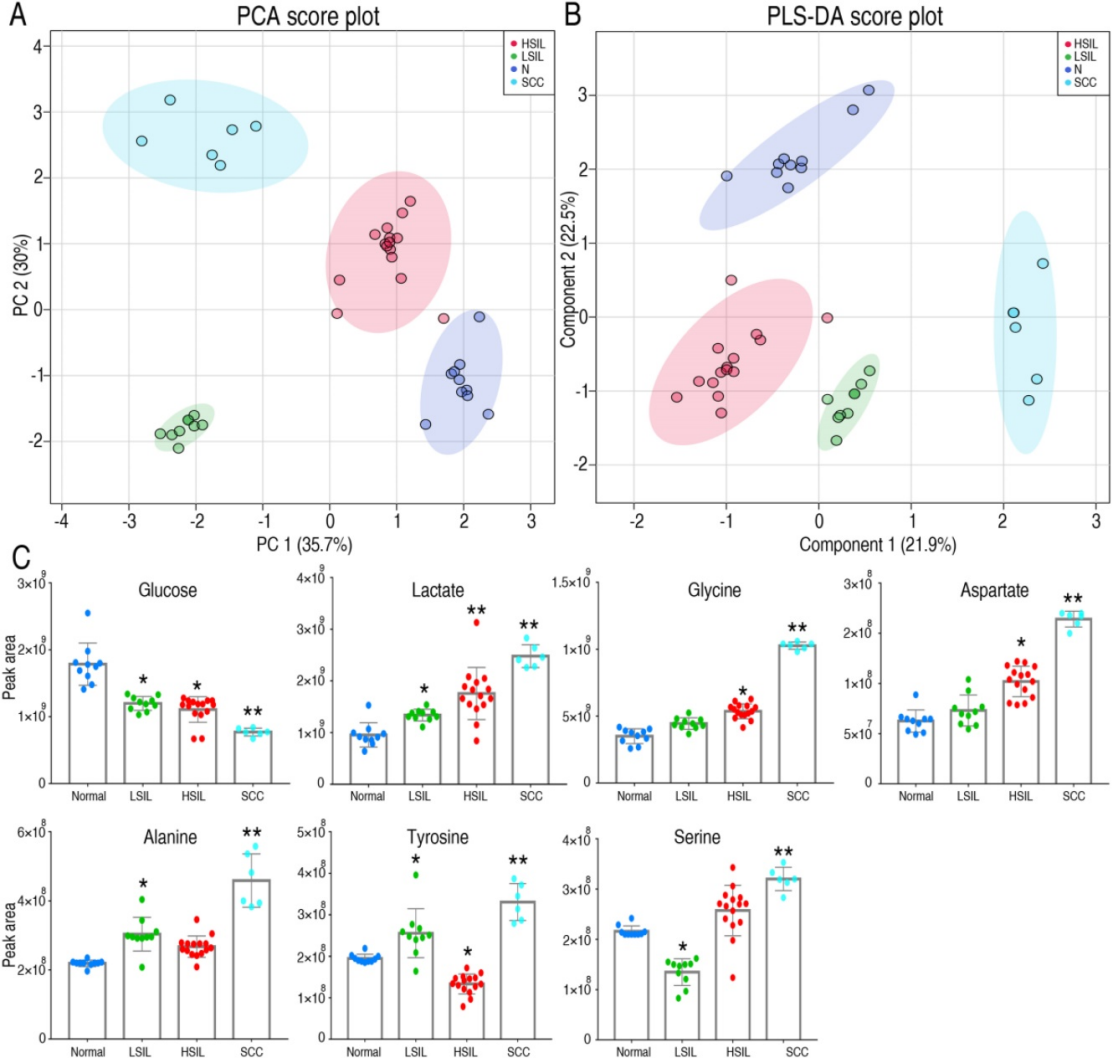
Metabolomics characteristics of cervical epithelial tissues. PCA and PLS-DA completely separated cervical precancerous lesions (HSIL and LSIL) from normal cervical epithelial tissues (N, Normal) and cervical cancer tissues (SCC) (A and B). Levels of several key metabolites in different cervical tissues were detected by GC-MS (C). LSIL, low-grade squamous intraepithelial lesions; HSIL, high-grade squamous intraepithelial lesions. *P < 0.05 vs Normal and **P < 0.01 vs Normal.

Metabolic changes in precancerous tissues were further verified. Metabolic profiles of cervical precancerous tissues were analyzed as described above, and compared with those of normal cervical epithelium and squamous cell carcinoma (SCC) (Figure S2). Twenty-three metabolites with reliable signals were identified (Table S1). PCA and PLS-DA analyses completely distinguished cervical precancerous lesions (LSIL and HSIL) from normal cervical epithelium and cervical cancer (Figure 5A, 5B). Interestingly, the comparison of peak areas of metabolites indicated that glucose consumption and lactate production increased from LSIL to the peak at HSIL and SCC compared with those of normal cervical epithelium (Figure 5C). The content of several amino acids, such as glycine, aspartate, alanine, tyrosine and serine, increased in SCC (Figure 5C). An increase of glycine, aspartate, alanine and serine in HSIL and of alanine and tyrosine in LSIL was also observed. Most of these amino acids can be derived from the intermediates of the glycolytic pathway.

### Expression of glycolytic enzymes and proteins changed in cervical precancerous lesions

**Figure 6:**
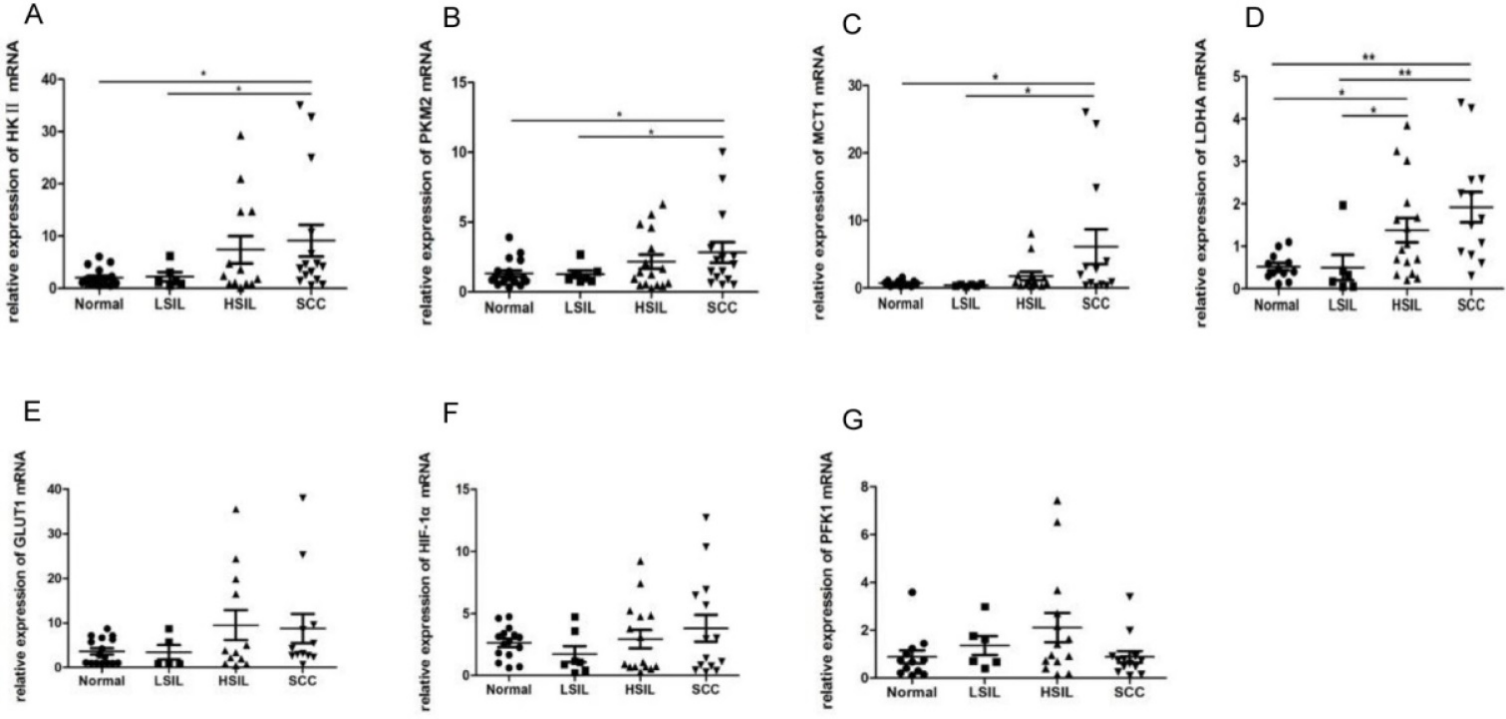
RT-PCR was used to detect the expression of HK II, PKM2, LDHA, MCT1, GLUT1, HIF-1α and PFK1 in cervical biopsies, including 15 normal cervical epithelial tissues, 6 low-grade squamous intraepithelial lesions (LSIL), 14 high-grade squamous intraepithelial lesions (HSIL) and 13 squamous cell carcinomas (SCC).^ *^P < 0.05, ^**^P < 0.01.

Enzymes and proteins such as HK Ⅱ, PFK1, PKM2, LDHA, GLUT1, MCT1 and HIF-1α are closely associated with glycolysis. The mRNA levels of these proteins in 48 cervical biopsies, including 15 normal cervical epithelia, 6 LSIL, 14 HSIL and 13 SCC, were detected by RT-PCR. The levels of HK Ⅱ, PKM2 and MCT1 in SCC were higher than those in normal cervical epithelia and LSIL (*P*<0.05) (Figure 6A, 6B, 6C). Whilst the expression of LDHA in SCC was significantly up-regulated compared with that in normal cervical epithelia and LSIL (*P* < 0.01), the level of LDHA in HSIL was also higher than that in normal cervical epithelia and LSIL (*P* < 0.05) (Figure 6D), suggesting that the expression of some glycolytic enzymes was deregulated in cervical precancerous lesions. The expression of GLUT1, PFK1 and HIF-1α showed no statistically significant change in different tissues (Figure 6E-G).

**Figure 7:**
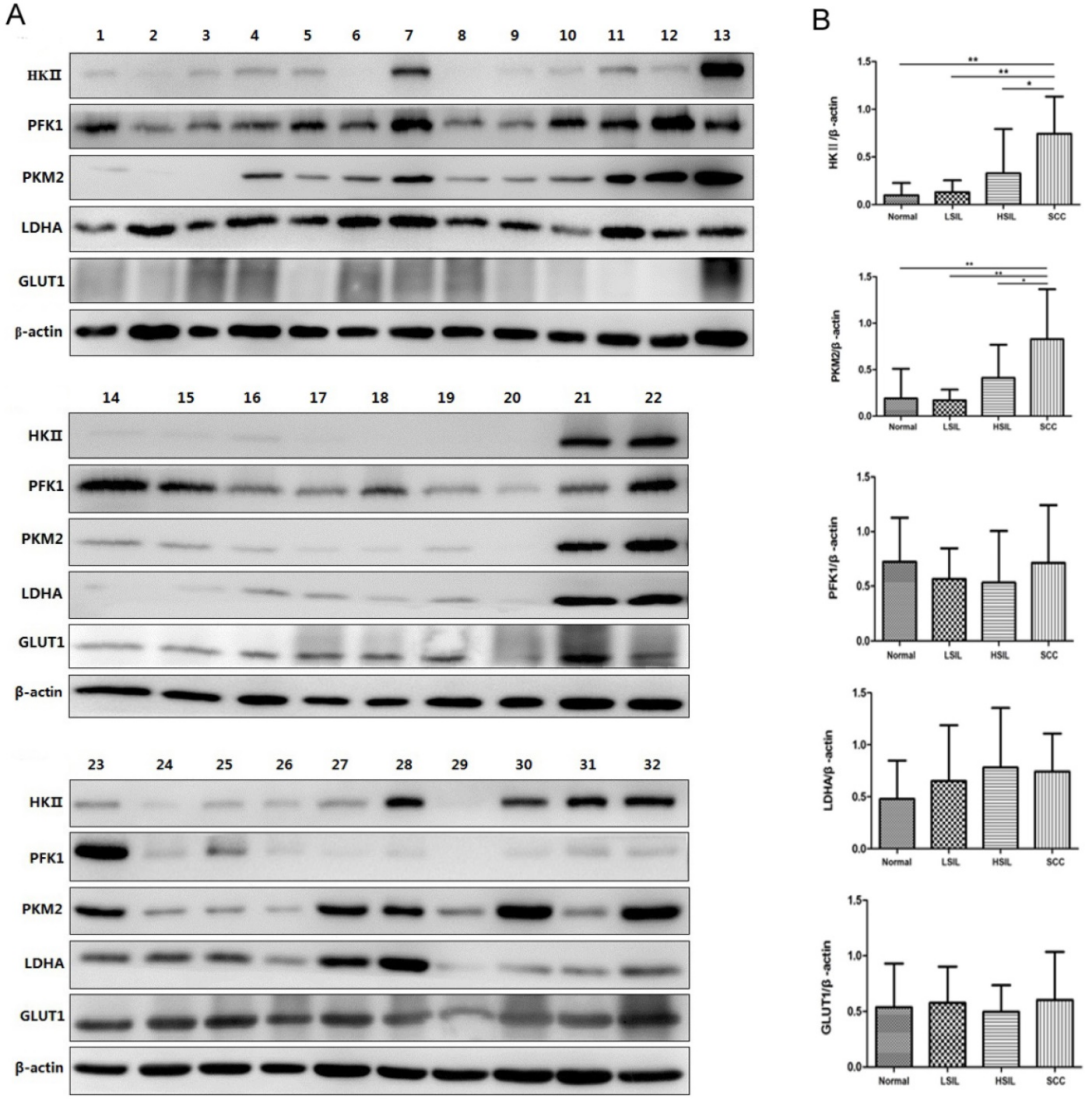
Expression of HK II, PFK1, PKM2, LDHA and GLUT1 in cervical biopsies was detected by Western blot analysis. In total, 32 biopsies were examined, including normal cervical tissues (samples 1, 2, 3, 14, 15, 16, 17, 23, 24), LSIL (samples 4, 5, 6, 18, 19, 25, 26), HSIL (samples 7, 8, 9, 10, 20, 27, 28, 29) and SCC (samples 11, 12, 13, 21, 22, 30, 31, 32) (A). The relative expression of HK II, PFK1, PKM2, LDHA and GLUT1 was normalized by β-actin (B). ^*^P < 0.05, ^**^P < 0.01.

Western blot analysis was further used to confirm the expression of HK Ⅱ, PFK1, PKM2, LDHA and GLUT1 in 32 cervical biopsies, including 9 normal cervical epithelia, 7 LSIL, 8 HSIL and 8 SCC (Figure 7A). The gray values of protein bands were analyzed according to the results of Western blot analysis. The levels of HK Ⅱ and PKM2 in SCC were higher than those in normal cervical epithelia and cervical precancerous lesions (Figure 7B), which were consistent with RT-PCR results. The expression of HK II and PKM2 in precancerous tissues also showed an increased trend compared with normal cervical epithelia (Figure 7B). The expression levels of PFK1, LDHA and GLUT1 were not significantly different among the 4 groups analyzed.

## Discussion

Metabolic reprogramming characterized by the Warburg effect is one of the hallmarks of cancer. In this study, the metabolome of human oral precancerous cell and cervical precancerous tissues was analyzed by GC-MS, and 115 metabolites in cell samples and 23 metabolites in tissue specimens were identified. By comparison of the metabolomes of these cells, the oral cancer cells could be clearly distinguished from the DOK and HOEC cells. Four metabolites (pyruvate, glutamine, methionine and lysine) were identified as critical differential molecules between DOK and HOEC. Glucose consumption and lactate production increased in the cervical squamous intraepithelial lesions (LSIL and HSIL), a kind of human cervical precancerous lesion. Some amino acids such as glycine and aspartate increased in cervical precancerous tissues (HSIL). The expression of several glycolysis-related enzymes and proteins such as HK II, PKM2 and MCT1 increased in cervical squamous cell carcinoma, and the expression of LDHA, HK II and PKM2 increased, to some extent, in cervical precancerous lesions.

It is well-known that cancer cells maintain a high glycolytic flux to meet the energy and intermediates needed for survival and rapid growth 2, 18. Compared with adjacent non-tumor tissues, many glycolytic enzymes are up-regulated in tumors 19. Recent studies have also shown the metabolic characteristics and preferences of a tumor change during tumor progression 20, 21. In fact, metabolic alteration in the precancerous stage or role of metabolic reprogramming during carcinogenesis has not been well-understood. Several investigators have investigated metabolic changes in the precancerous stage and found some preliminary signs of metabolic reprogramming in premalignant cells or tissues. For example, an early imaging study showed increased glycolysis and glutamine consumption in precancerous epithelial tissues 22. Detection of the metabolism-related genes in colorectal biopsies revealed that the expression of HIF-1α, GLUT1, PKM2 and LDHA in precancerous colorectal lesions was significantly higher than that in normal controls, which provided evidence for early Warburg-like metabolic changes in premalignant colorectal mucosa 23. Transcriptome analysis in a familial adenomatous polyposis (FAP) model confirmed that glucose metabolism was reprogrammed in the precancerous adenoma stage 24. A shift from oxidative phosphorylation to glycolysis was found in the very early stage of hepatocarcinogenesis in a rat model 25. Glycolytic enzymes such as HK Ⅱ, PKM2 and aldolase A were up-regulated were up-regulated in precancerous cirrhotic livers, which is closely related to an increased risk of hepatocellular carcinoma 19. The metabolites of glycolysis and the pentose phosphate pathway were up-regulated in the early stages of pancreatic cancer in a mouse model 8. However, metabolomic evidence for the Warburg effect in precancerous lesions remains limited. In this study, we found that the level of pyruvate increased in oral precancerous cells, and glucose consumption and lactate production were enhanced in cervical precancerous lesions. Some glycolytic enzymes such as LDHA increased in cervical precancerous lesions as well. Our findings, including metabolites and related genes, further support metabolic reprogramming to the Warburg effect in precancerous lesions.

Metabolic reprogramming for cancer also involves the metabolism of amino acids, characterized by increased consumption of glutamine 1. Increased biosynthesis of some amino acids may even contribute to cell transformation and tumorigenesis 26. In this study, glutamine, methionine and lysine were considered to be important differential amino acids between DOK and HOEC cells, and glycine and aspartate increased in HSIL tissues, suggesting that amino acid metabolism may play an important role in the development of squamous epithelial lesions or progression of epithelial lesions to invasive carcinomas. These amino acids have been associated with dysplasia or cancer. Increasing glutamine by reprogramming glutamine metabolism promotes nucleotide biosynthesis and liver cancer formation 27. It was found that tumor-initiating cells (TICs) are metabolically dependent on methionine. The methionine cycle activity of these cells was highly enhanced, and inhibition of the methionine cycle was enough to damage the tumor-initiating capability of TICs 28. Dietary restriction of methionine inhibited tumor growth and made tumor sensitive to chemotherapy by disrupting one-carbon metabolism 29. Glycine can produce one-carbon units and integrate into the purine ring, and *de novo* synthesis of glycine can fuel purine nucleotide biosynthesis in tumor tissues 30, 31. It has been reported that the serine, glycine, one-carbon (SGOC) metabolic network was up-regulated in neuroendocrine prostate cancer 32, 33. The content of aspartate in breast cancer tissues and cells was significantly higher than that in adjacent non-tumor tissues and breast epithelial cells, suggesting that tumor increased the utilization of aspartate 34. The level of aspartate and its downstream metabolites was up-regulated when prostate cancer cells underwent epithelial mesenchymal transition (EMT) 35. Aspartate is considered to be a limiting metabolite for tumor growth 36, 37. Enhanced glycolysis can also provide intermediates for the biosynthesis of amino acids. For example, glycine can be derived from 3-phosphoglycerate.

Although a few studies have found that metabolic alterations occur in precancerous cells and contribute to the development of cancer, their exact significance and mechanisms in tumorigenesis remain open questions. In this study, we revealed that the metabolism of glucose and amino acids has been reprogrammed in precancerous epithelial cells and tissues. We noted that the metabolic changes in oral precancerous cell lines and in cervical precancerous lesions were not identical. That makes sense. The heterozygosity of tumors is great, and the pathogenesis of different tumors is also diversified. The status of metabolic reprogramming in different tumor should be different. In addition, some results obtained by RT-PCR were not exactly consistent with those achieved in Western blot analysis. The sample size might have been a contributing factor. Our findings provide potential evidence for the Warburg-like effect in precancerous lesions. Further studies are needed to evaluate or verify this phenomenon.

## Supplementary Material

Supplementary figures and tables.
