# Supplementary material for: Metabolomics study reveals the potential evidence of metabolic reprogramming towards the Warburg effect in precancerous lesions
Source: J Cancer. 2021 Jan 10;12(5):1563–74. doi: 10.7150/jca.54252 (PMC7847643; doi:10.7150/jca.54252)
Supplement: Supplementary file 1 — Supplementary figures and tables. [file jcav12p1563s1.pdf]

Supplementary materials

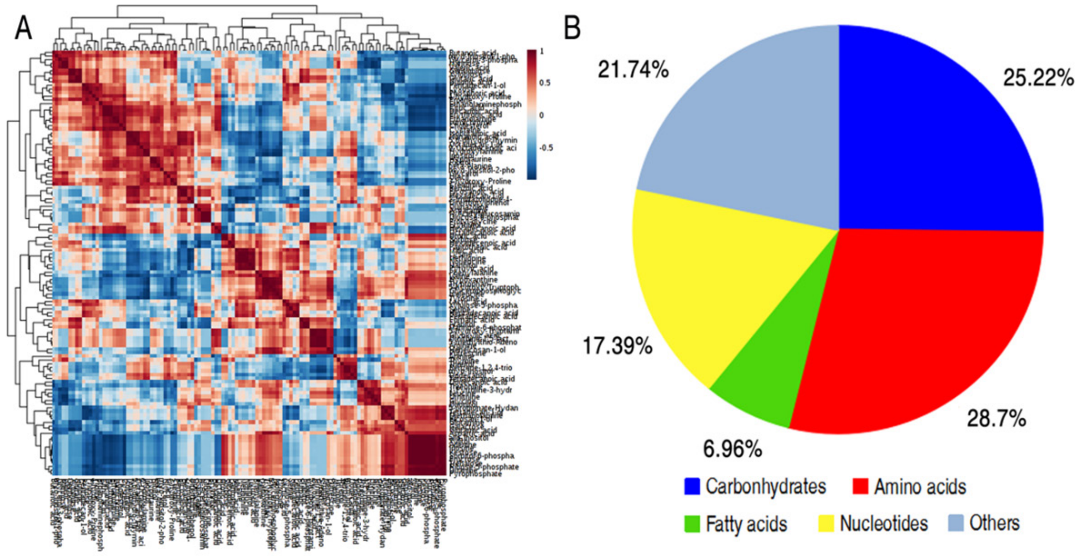

Figure S1. Reproducibility of the metabolomics profiling platform (A) and metabolite distribution (B). The Figure shows metabolite abundance quantified in cell samples over two technical replicates. Correlation coefficients between technical replicates varied between 0.9799 and 0.9999. This map shows the two replicates with a correlation of more than 0.9799. The identified metabolites were categorized according to KEGG (http://www.kegg.jp/) and NCBI PubChem (https://pubchem.ncbi.nlm.nih.gov/).

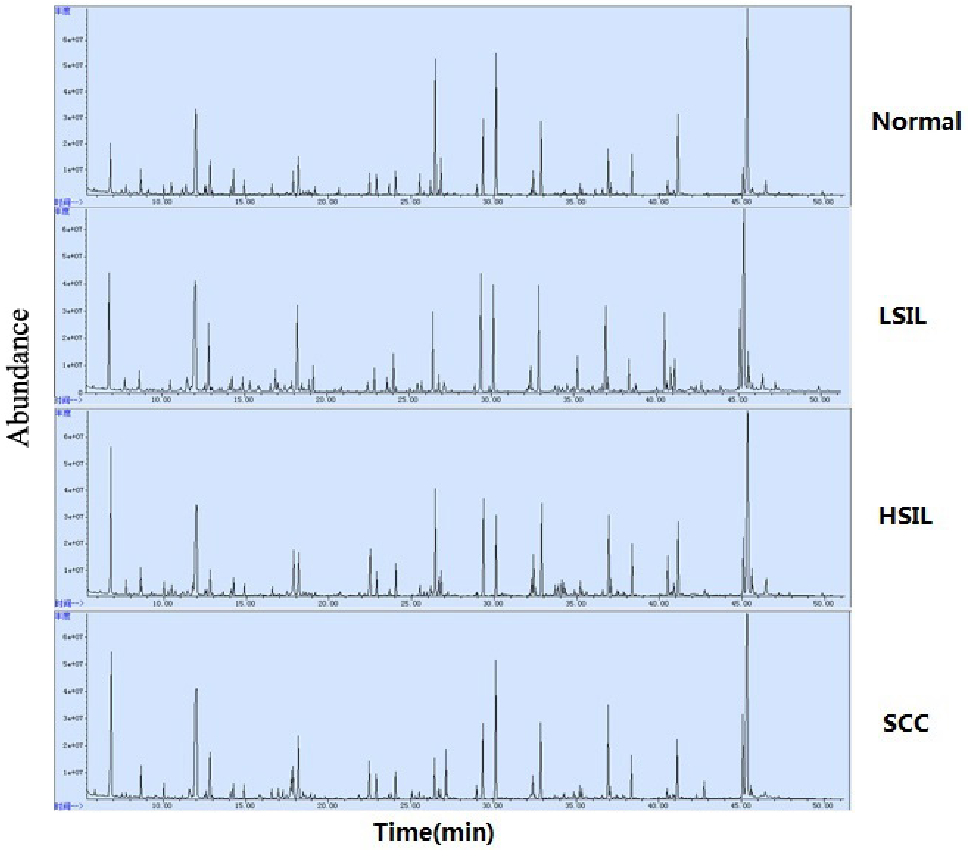

Figure S2. Metabolomics profiles of different cervical tissues. Representative total ion current chromatograms are shown. Normal, normal cervical epithelial tissues; LSIL, low-grade squamous intraepithelial lesions; HSIL, high-grade squamous intraepithelial lesions; and SCC, squamous cell carcinomas. X-axis, retention time (min); Y-axis, abundance.

Table S1. Relative abundance of metabolites in different cervical tissues determined by GC-MS.

Metabolites	RT (min)	Peak area (X±SD), ×10^8				*P*	*P*	*P*
		Normal	LSIL	HSIL	SCC	N vs LSIL	N vs HSIL	N vs SCC
Lactate	6.851	8.74±5.57	8.88±5.26	23.12±13.23	21.99±7.42	0.431	0.007	0.010
Valine	7.447	1.29±0.26	1.02±0.15	1.58±0.17	1.41±0.32	0.158	0.148	0.530
Alanine	7.736	1.78±0.20	2.64±0.47	3.05±0.98	3.91±0.93	0.148	0.041	0.003
Phosphate	7.949	2.39±1.85	2.68±1.86	3.27±1.16	5.87±1.67	0.819	0.494	0.017
Leucine	9.017	0.14±0.07	0.19±0.11	0.35±0.17	0.42±0.25	0.731	0.140	0.058
Urea	11.166	1.86±1.32	2.23±1.99	2.68±1.11	1.99±0.31	0.882	0.434	0.900
Isoleucine	12.518	0.90±0.20	0.84±0.04	0.96±0.23	0.86±0.05	0.524	0.608	0.705
Proline	12.612	4.98±3.74	0.45±0.25	10.57±6.44	0.72±0.20	0.079	0.036	0.096
Glycine	12.843	3.24±1.04	3.74±0.87	5.80±1.08	7.55±1.59	0.591	0.017	0.001
Pyrimidine	13.610	0.16±0.09	0.25±0.09	0.63±0.42	0.73±0.42	0.690	0.069	0.032
Fumarate	14.006	0.12±0.00	0.15±0.10	0.29±0.19	0.29±0.18	0.857	0.316	0.332
Serine	14.248	1.76±0.12	1.12±0.37	3.21±1.19	2.56±0.95	0.293	0.030	0.195
Aspartate	15.865	0.53±0.14	0.55±0.24	1.00±0.28	0.81±0.15	0.885	0.010	0.088
Succinate	17.459	0.43±0.09	0.69±0.41	1.18±0.61	0.88±0.35	0.460	0.053	0.213
Cysteine	19.006	0.20±0.00	0.08±0.03	0.22±0.10	0.15±0.01	0.107	0.868	0.433
Uridine	23.811	0.66±0.17	0.38±0.34	0.32±0.15	0.19±0.03	0.239	0.153	0.066
Fructose	25.942	0.07±0.02	0.08±0.04	2.86±1.57	2.66±1.50	0.996	0.162	0.181
Tyrosine	26.161	1.60±0.20	2.21±0.61	1.67±0.52	2.84±0.58	0.147	0.861	0.009
Glucose	26.231	16.03±6.84	9.04±1.05	9.05±6.28	4.22±1.34	0.671	0.005	0.004
Galactose	26.314	0.00±0.00	0.19±0.09	0.37±0	1.59±0.67	0.781	0.585	0.08
Arachidonate	34.868	0.36±0.30	0.33±0.10	1.18±0.48	1.62±0.50	0.919	0.016	0.001
Maltose	40.500	2.50±1.12	15.31±2.91	9.74±7.11	4.25±3.74	0.078	0.661	0.953
Lactose	41.492	1.56±0.00	1.57±0.00	0.61±0	0.59±0.00	0.674	0.856	0.843

HSIL, high-grade squamous intraepithelial lesions; LSIL, low-grade squamous intraepithelial lesions; Normal (N), normal cervical epithelial tissues; RT, retention time; SCC, squamous cell carcinomas; and SD, standard deviation.
